# Detection of *Trypanosoma cruzi* in the saliva of diverse neotropical bats

**DOI:** 10.1111/zph.12808

**Published:** 2021-01-23

**Authors:** Laura M. Bergner, Daniel J. Becker, Carlos Tello, Jorge E. Carrera, Daniel G. Streicker

**Affiliations:** ^1^ Institute of Biodiversity, Animal Health and Comparative Medicine College of Medical, Veterinary and Life Sciences University of Glasgow Glasgow UK; ^2^ MRC–University of Glasgow Centre for Virus Research Glasgow UK; ^3^ Department of Biology University of Oklahoma Norman OK USA; ^4^ Association for the Conservation and Development of Natural Resources Lima Perú; ^5^ Yunkawasi Lima Perú; ^6^ Departamento de Mastozoología Museo de Historia Natural Universidad Nacional Mayor de San Marcos Lima Perú; ^7^ Programa de Conservación de Murciélagos de Perú Piura Perú

**Keywords:** Chiroptera, *Desmodus rotundus*, protozoa, shotgun metagenomics, wildlife, zoonoses

## Abstract

*Trypanosoma cruzi* is widely reported in bats, yet transmission routes remain unclear. We present evidence from metagenomic sequence data that *T. cruzi* occurs in the saliva of diverse Neotropical bats. Phylogenetic analyses demonstrated that the bat‐associated *T. cruzi* sequences described here formed part of a bat‐specific clade, suggesting an independent transmission cycle. Our results highlight the value in repurposing metagenomic data generated for viral discovery to reveal insights into the biology of other parasites. Evaluating whether the presence of *T. cruzi* in the saliva of two hematophagous bat species represents an ecological route for zoonotic transmission of Chagas disease is an interesting avenue for future research.

**Impacts**

- Chagas disease caused by *Trypanosoma cruzi* affects millions of people, but the dynamics of parasite transmission within sylvatic cycles remain poorly known
- We report the presence of *T. cruzi* I in the saliva of four Neotropical bat species, which phylogenetic analyses suggested represented a bat‐specific transmission cycle
- *T. cruzi* I was detected in two hematophagous bat species, underlining the need for further research into the potential risk of zoonotic transmission directly from bat bites

## 1 INTRODUCTION

Chagas disease, caused by the parasite *Trypanosoma cruzi*, affects over 6 million people, mostly in the Americas. Infections in humans can cause acute febrile illness in 1%–5% of individuals, while an estimated 20%–30% of infections can transition into a chronic disease associated with cardiac disorders and sudden death (Bern, 2015; Shikanai‐Yasuda & Carvalho, 2012). Human infections predominately arise in domestic or peridomestic cycles of stercorarian transmission from triatomine vectors; however, alternative transmission routes of *T. cruzi* can include transfusion and transplantation (Bern, 2015; Perez‐Molina & Molina, 2018). In light of successful vector control programs and serological screening in blood banks to prevent transfusions of infected blood, congenital transmission and orally transmitted infections originating from sylvatic cycles are of increasing epidemiological importance (Perez‐Molina & Molina, 2018; Shikanai‐Yasuda & Carvalho, 2012). Here, we focus on sylvatic cycles of *T. cruzi* in wildlife, which can be maintained in animal populations through vector‐borne transmission, consumption of contaminated material, or predation on infected hosts or vectors (Jansen et al., 2015). Additionally, some wildlife species such as opossums experimentally and naturally maintain multiple parasite life stages (Barros et al., 2020; Deane et al., 1984) and have been hypothesized to transmit *T. cruzi* in the absence of arthropod vectors (Shikanai‐Yasuda et al., 1991; Urdaneta‐Morales & Nironi, 1996). The recent detection of *T. cruzi* in the salivary glands of *Diaemus youngi*, a hematophagous bat, suggests the possibility that bats could also act as both reservoirs and transmitters of the parasite (Villena et al., 2018). Bats are important trypanosome reservoirs which host both generalist and bat‐restricted trypanosomes (Marcili et al., 2009; Ramírez et al., 2014) and have been suggested as the ancestral host of trypanosomes (Hamilton et al., 2012). Identifying routes of trypanosome transmission in bats may shed new light on sylvatic cycles of the parasite and inform strategies to reduce zoonotic transmission.

## 2 MATERIALS AND METHODS

**FIGURE 1:**
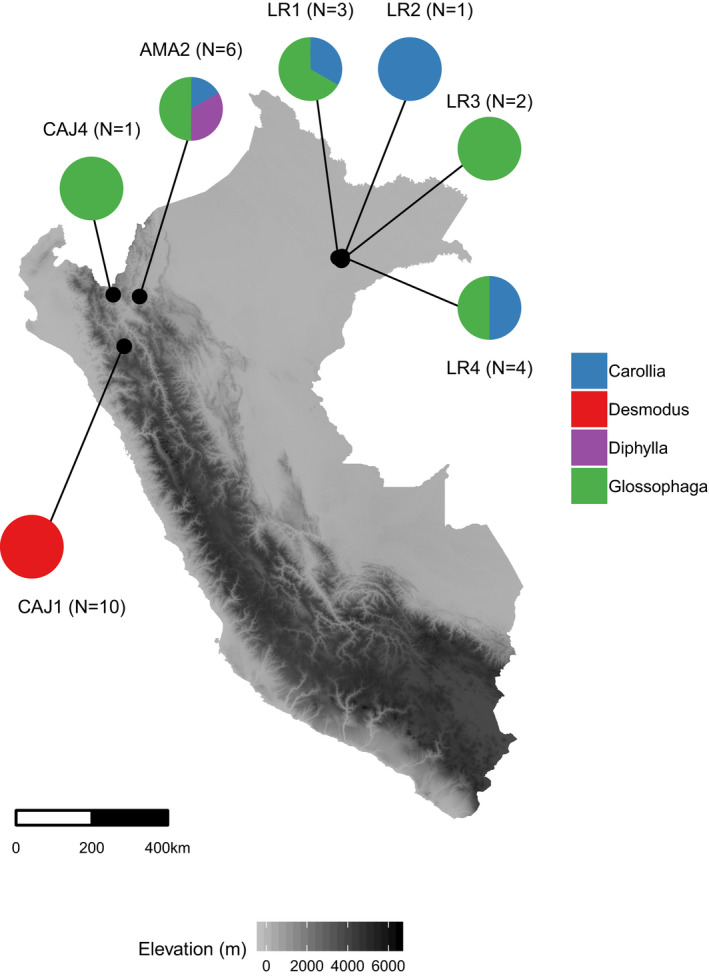
Sampling of bats in Peru. Circles show the proportion of individuals of a given bat genus captured at each site, and the total sample size is listed beside the site name. Individuals of the same species were combined across sites into one pool for metagenomic sequencing

As part of a virus discovery project, in 2016, we captured bats across seven sites in northern Peru (Departments of Amazonas, Cajamarca and Loreto) using mist nets, harp traps and hand nets (Figure 1) (Bergner et al., 2020). Samples were collected from four bat species (*N* = 27 individuals total) representing frugivores (*Carollia perspicillata,* *N* = 10), nectarivores (*Glossophaga soricina*, *N* = 5) and two sanguivores (*Desmodus rotundus,* *N* = 10 and *Diphylla ecaudata,* *N* = 2) specializing on mammals and birds, respectively. Sampling protocols were approved by the Research Ethics Committee of the University of Glasgow School of Medical, Veterinary and Life Sciences (Ref081/15), the University of Georgia Animal Care and Use Committee (A2014 04–016‐Y3‐A5), and the Peruvian Government (RD‐142–2015‐SERFOR‐DGGSPFFS, RD‐054–2016‐SERFOR‐DGGSPFFS).

Saliva was collected using sterile cotton‐tipped swabs (Fisherbrand) which were stored in 1ml RNALater (Ambion) overnight at 4°C then transferred to −80°C. Total nucleic acid was extracted from individual swabs using a KingFisher Flex 96 (Thermo) and a BioSprint One for All Vet Kit (Qiagen) (Bergner et al., 2019). Extracts were pooled by bat species (Table 1) and depleted of host material using DNAse (Bergner et al., 2019). Libraries were prepared for untargeted metagenomic sequencing using the Clontech SMARTer Stranded Total RNA‐Seq Kit v2 (Takara), then sequenced on an Illumina NextSeq500 at the University of Glasgow Polyomics Facility. Sequencing reads (European Nucleotide Archive project PRJEB35111) were processed using an in‐house bioinformatic pipeline (Bergner et al., 2019), with slight modification to the read trimming step to accommodate the library preparation kit and read length.

**TABLE 1:** Summary of Neotropical bat saliva metagenomic sequencing

Species	Individuals in pool	Raw reads	*Trypanosoma*‐like reads	*Trypanosoma*‐like contigs	cytB accession	gGAPDH accession
*Glossophaga soricina*	5	24,079,752	347,241	20,302	MT572485	MT572489
*Diphylla ecaudata*	2	25,023,095	100,377	2,532	MT572486	—
*Desmodus rotundus*	10	28,946,275	113,219	2,752	MT572487	MT572490
*Carollia perspicillata*	10	28,700,978	18,328	293	MT572488	—

The pipeline used SPAdes v.3.10.1 (Bankevich et al., 2012) for de novo assembly and Diamond v.0.8.20 blastx (Buchfink et al., 2014) for classification of contigs, which revealed *Trypanosoma*‐like Cytochrome B (cytB) sequences in all pools and *Trypanosoma*‐like glycosomal glyceraldehyde 3‐phosphate dehydrogenase (gGAPDH) sequences in two of four pools (Table 1). Representative sets of *T. cruzi* cytB and gGAPDH sequences from different hosts and vectors (Table S1 and Table S2) were aligned with new *T. cruzi* sequences from bats using MAFFT 7.017 (Katoh et al., 2002) within Geneious 7.1.7 (Kearse et al., 2012). For both genes, we focused on regions present in novel and published sequences, using trimal with automatic parameters (Capella‐Gutiérrez et al., 2009) on the Phylemon server (Sanchez et al., 2011) to remove alignment ends with missing data across most samples. There were no internal alignment gaps present in regions analysed, such that end trimming left reading frames intact. Both cytB and gGAPDH datasets were restricted to unique sequences, with the exception of sequences from *T. cruzi* in bat saliva and other bat‐associated TcI sequences.

For each alignment, the best model of sequence evolution and support for codon partitioning were evaluated using PartitionFinder2 (Lanfear et al., 2017) on the CIPRES Science Gateway 3.3, which was run with linked branch lengths, the greedy search algorithm, and BIC criterion. For the cytB analysis, PartitionFinder supported codon partitioning with the models HKY + G, F81 and GTR + G applied to the first, second and third codon positions, respectively. For the gGAPDH analysis, PartitionFinder indicated the models JC, HKY and F81 applied to the first, second and third codon positions, respectively. Bayesian phylogenetic analysis of cytB and gGAPDH was performed using MrBayes 3.2.6 (Ronquist et al., 2012) on the CIPRES server with the substitution models and partitioning scheme indicated by PartitionFinder. Each analysis was run for 2,000,000 generations and sampled every 2,000 generations, with the first 20% of trees discarded as burn‐in. Maximum likelihood phylogenetic analysis of cytB and gGAPDH was conducted using RAxML 8.2.8 (Stamatakis et al., 2008). As RAxML only allows a single model of rate heterogeneity in partitioned analysis, separate PartitionFinder analyses were run for each type of rate heterogeneity. The scheme with lowest BIC score was selected for each alignment, yielding the substitution model GTR + G for cytB and GTR for gGAPDH. RAxML was then run with 1,000 bootstrap replicates using the indicated substitution model and codon partitioning. Figures were prepared in R version 3.5.3 (R Core Team, 2019) using the packages ‘ape’ (Paradis & Schliep, 2019), ‘phangorn’ (Schliep, 2010), ‘phytools’ (Revell, 2011) and ‘ggtree’ (Yu et al., 2016).

## 3 RESULTS AND DISCUSSION

**FIGURE 2:**
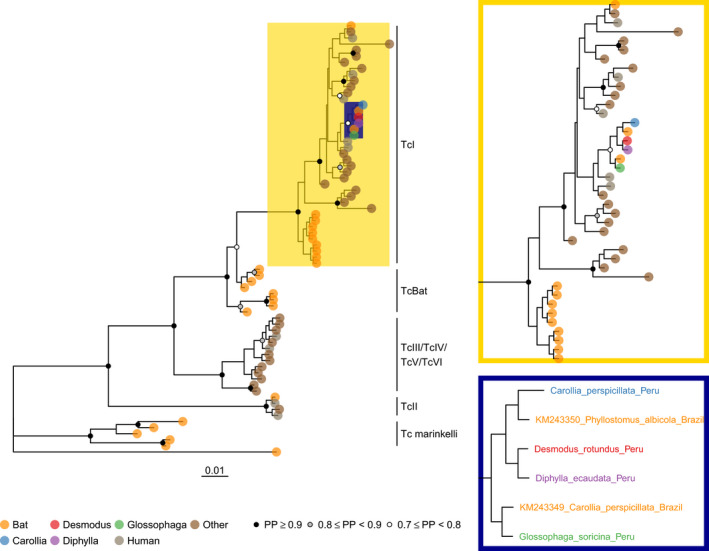
*Trypanosoma cruzi* cytB phylogeny. The phylogeny was constructed in MrBayes based on a 476bp alignment of 71 *Trypanosoma* cytochrome B sequences, rooted on *Trypanosoma dionisii* (Genbank accession FJ900249). The TcI lineage and the bat sub‐clade are highlighted in gold and blue, respectively, and expanded for further detail

Sequences matching the genus *Trypanosoma* were abundant in all bat species tested (18,328–347,241 reads per pool; Table 1). Bayesian and Maximum Likelihood phylogenetic analysis of cytB and gGAPDH classified all novel bat‐associated sequences within the *T. cruzi* TcI lineage (Figure 2; Figure S1; Figure S2). Although the Peruvian bat‐derived sequences did not group together in the gGAPDH phylogeny, likely due to lack of sequence variation, cytB sequences clustered with TcI sequences from Brazilian bats (Lima et al., 2014) (posterior probability = 0.77; bootstrap support = 58%). Other Neotropical bat‐derived TcI sequences from Venezuela, Colombia and Brazil were dispersed amongst non‐bat TcI samples or formed a distinct bat‐associated clade towards the base of the TcI lineage (Figure 2; Figure S1), as observed previously (Marcili et al., 2009). Sequences from bat and non‐bat hosts did not cluster together for any country where both were available (i.e., Venezuela, Colombia, Brazil), demonstrating that geographic structure alone does not explain the occurrence of bat‐associated TcI clades (Table S1). TcI has been hypothesized to have its origins in marsupials due to high levels of strain diversity in these hosts (Brenière et al., 2016), but it also occurs in diverse bat species (Lima et al., 2014; Marcili et al., 2009; Ramírez et al., 2014). Our results support the conclusion that bats can maintain independent transmission cycles of this lineage. Although our approach focused only on TcI, future studies could employ metabarcoding (e.g., Dario et al., 2017) to explore the diversity of other *Trypanosoma* species present in bat saliva. More generally, as our data were originally generated for virus discovery, we show how metagenomic data can simultaneously reveal insights into diverse pathogens.

The discovery of *T. cruzi* in bat saliva has several plausible ecological explanations with different implications for transmission. Since the four infected bat species have different feeding behaviours, a common source of dietary contamination is unlikely. Given the expected role of arthropods in *T. cruzi* transmission, presence in saliva might arise from inadvertent consumption of ectoparasites while grooming. This hypothesis is supported by the observation that bat‐associated ectoparasites in the family Cimicidae experimentally replicate and transmit other *Trypanosoma* species (Gardner & Molyneux, 1988). Oral infection of humans by a similar route further supports the viability of this transmission mode (Shikanai‐Yasuda & Carvalho, 2012). Alternatively, *T. cruzi* may be excreted in bat saliva, as supported by infection in the salivary glands of another hematophagous bat species, *D. youngi* (Villena et al., 2018). If verified, bat‐to‐bat transmission in the absence of arthropods would represent a novel transmission route which might occur through social contacts, biting, or—in the case of *D. rotundus*—blood‐meal sharing.

Although *T. cruzi* has been documented in the salivary glands of *D. youngi* (Villena et al., 2018), our findings comprise the first evidence of TcI in the saliva of *D. rotundus* and *D. ecaudata*, two vampire bat species which are known to feed on humans (Ito et al., 2016). Notably, the area of northern Peru where our study was conducted is a hotspot for vampire bat depredation on humans which has been associated with recurrent rabies outbreaks (Gilbert et al., 2012; Stoner‐Duncan et al., 2014). The hematophagous diet of *D. rotundus* therefore provides an ecological route for *T. cruzi* transmission to diverse non‐bat mammals, including humans.

Ultimately, the likelihood of zoonotic transmission will be determined by the viability of infectious parasites in bat saliva. Since parasite viability cannot be evaluated using metagenomic data, isolation of the parasite and establishing the presence of metacyclic trypomastigotes are crucial next steps to evaluate zoonotic risk. In addition, parasite load is an important determinant of infection for other transmission modes (e.g., congenital; Bustos et al., 2019), but our sequencing approach of pooling DNA from multiple individuals precludes any such quantification. Efforts to accurately quantify parasite load in saliva, using methods such as quantitative PCR, would be valuable. Zoonotic transmission also depends on the susceptibility of humans to bat‐associated strains. In our study, the cytB and gGAPDH phylogenies suggest that the parasites detected in bats belong to the TcI lineage of *T. cruzi,* which is generally assumed to be capable of infecting humans. However, we note that multi‐locus sequence typing and 18S ribosomal RNA sequencing can more sensitively discriminate *T. cruzi* lineages, so additional sequencing of these markers is needed to confirm the identity of trypanosomes as *T. cruzi* I (Dario et al., 2017; Yeo et al., 2011). This is particularly relevant given that our sequences represent a consensus based on pools made up of multiple individuals, and others have reported a high frequency of mixed infections even in individuals bats (Dario et al., 2017; Jansen et al., 2018).

In conclusion, our study reports likely bat‐maintained transmission cycles of the TcI lineage of *T. cruzi* and possible shedding of these parasites in the saliva of two bat species which can feed on humans. The origins and implications of *T. cruzi* DNA in bat saliva provide an interesting avenue for further research. Given the regional significance of Chagas disease, it is important to evaluate the risk posed by bats as both reservoirs and transmitters of zoonotic trypanosome infections.

## CONFLICT OF INTEREST

The authors declare no conflict of interest. The funders had no role in the design of the study; in the collection, analyses or interpretation of data; in the writing of the manuscript, or in the decision to publish the results.

## Supporting information

Supplementary Material

## Data Availability

Metagenomic sequence data are available on the European Nucleotide Archive (Project PRJEB35111 https://www.ebi.ac.uk/ena/browser/view/PRJEB35111) and *Trypanosoma* sequences are available on Genbank (Accessions MT572485‐MT572490).
