# Supplementary material for: Detection of Trypanosoma cruzi in the saliva of diverse neotropical bats
Source: Zoonoses Public Health. 2021 Jan 23;68(3):271–6. doi: 10.1111/zph.12808 (PMC8569697; doi:10.1111/zph.12808)
Supplement: Supplementary file 1 — Supplementary Material [file ZPH-68-271-s001.pdf]

Supplementary Material for:

Detection of *Trypanosoma cruzi* in the saliva of diverse Neotropical bats

Laura M. Bergner*, Daniel J. Becker, Carlos Tello, Jorge E. Carrera & Daniel G. Streicker

* Author for correspondence: Laura.Bergner@glasgow.ac.uk

This file includes:

- Table S1-S2
- Figures S1-S2

Table S1. Details of sequences included in cytB phylogenetic analysis including Genbank accession, sample identification used within the original study, sampled host, and country.

Genbank accession	Isolate/Strain	Host	Country
MT572485	GS_SV_Peru_7.2	*Glossophaga soricina*	Peru
MT572486	DE_SV_Peru_7.4	*Diphylla ecaudata*	Peru
MT572487	DR_SV_Peru_7.3	*Desmodus rotundus*	Peru
MT572488	CP_SV_Peru_7.1	*Carollia perspicillata*	Peru
EU856369	TCCUSP269	*Saguinus midas*	Brazil
HQ713697	AAC1cl3	*Rhodnius prolixus*	Colombia
AJ130928	X10_cl1	*Homo sapiens*	Brazil
FJ549386	TryCC125	*Didelphis aurita*	Brazil
KF220740	LJVPcl7	*Homo sapiens*	Colombia
JX431284	ANT3P1C6	*Homo sapiens*	Venezuela
KF220731	XCHcl14	*Homo sapiens*	Colombia
FJ555646	TryCC1620	*Euryoryzomys macconnelli*	Brazil
FJ555639	TryCC1107	*Rhodnius stali*	Brazil
FJ555634	TryCC540	*Rattus rattus*	Venezuela
EU856370	TCCUSP331	*Cebus apella*	Brazil
JF267930	Hap3	*Mepraia spinolai*	Chile
AJ439719	Cuica	*Philander opossum*	Brazil
EU559323	A04F	*Mepraia spinolai*	Chile
FJ555637	TryCC884	*Panstrongylus megistus*	Brazil
JF267939	Hap12	*Triatoma infestans*	Chile
JQ581362	XE2929	*Didelphis marsupialis*	Brazil
EU559324	24tp18	*Capra aegagrus hircus*	Chile
FJ549391	TryCC640	*Carollia perspicillata*	Brazil
FJ549392	TryCC642	*Carollia perspicillata*	Brazil
FJ002255	TryCC417	*Thyroptera tricolor*	Brazil
FJ002256	TryCC507	*Carollia perspicillata*	Brazil
KC951586	SL7389123	Bat	Colombia
KC951575	N10	Bat	Colombia
KC951589	T23	Bat	Colombia
KC951583	N22	Bat	Colombia
KC951592	U21231	Bat	Colombia
KT305777	TCC1338	*Carollia perspicillata*	Venezuela
FJ183398	TryCC45	*Didelphis aurita*	Brazil
KM243349	JFV306	*Carollia perspicillata*	Brazil

KM243350	JFV307	*Phyllostomus albicola*	Brazil
EU559326	26tp18	*Capra aegagrus hircus*	Chile
EU559329	5p18	*Capra aegagrus hircus*	Chile
EU559327	58p18	*Octodon degus*	Chile
EU559328	54p18	*Octodon degus*	Chile
KT829466	MCQ1495	*Glossophaga soricina*	Ecuador
KT305785	TCC2476	*Artibeus lituratus*	Colombia
KT305782	TCC2471	*Artibeus lituratus*	Colombia
KT305778	TCC204	*Myotis albescens*	Brazil
FJ002253	TryCC203	*Myotis ruber*	Brazil
FJ002261	TryCC1122	*Myotis albescens*	Brazil
FJ002257	TryCC597	*Myotis nigricans*	Brazil
FJ002259	TryCC947	*Myotis nigricans*	Brazil
HQ713711	Neoclicl6	*Rhodnius pallescens*	Colombia
FJ549388	TryCC185	*Triatoma infestans*	Bolivia
JQ581369	CM17	*Dasypus sp.*	Colombia
JF267935	Xd103	*Homo sapiens*	Chile
JQ581370	Saimiri3cl1	*Saimiri sciureus*	Venezuela
EU856372	TCCUSP668	*Rhodnius robustus*	Brazil
JF267936	Xd143	*Homo sapiens*	Chile
FJ549393	TryCC863	*Euphractus sexcinctus*	Brazil
FJ555631	TryCC132	*Philander frenatus*	Brazil
AJ439720	M5631	*Dasypus novemcinctus*	Brazil
FJ549396	TryCC1078	*Triatoma rubrovaria*	Brazil
EU856374	TCCUSP712	*Monodelphis brevicaudata*	Brazil
AJ130930	DogT	*Canis familiaris*	USA
AJ439727	Stc33R	*Procyon lotor*	USA
AJ439722	CBB	*Homo sapiens*	Chile
AJ130932	TU18_cl2	*Triatoma infestans*	Bolivia
AJ130931	Esmeraldo_cl3	*Homo sapiens*	Brazil
KT305789	TCC2557	*Phyllostomus hastatus*	Brazil
JN651278	24	Bat	Bolivia
JN543702	TCC501	*Carollia perspicillata*	Brazil
FJ900246	TryCC494	*Phyllostomus discolor*	Brazil
FJ900248	TryCC1089	*Artibeus planirostris*	Brazil
KT829468	MBC1539	*Artibeus fraterculus*	Ecuador
FJ900249	TryCC211	*Eptesicus brasiliensis*	Brazil

Table S2. Details of sequences included in gGAPDH phylogenetic analysis including Genbank accession, sample identification used within the original study, sampled host, and country.

Genbank accession	Isolate/Strain	Host	Country
MT572489	GS_SV_Peru_7.2	*Glossophaga soricina*	Peru
MT572490	DR_SV_Peru_7.3	*Desmodus rotundus*	Peru
KT305797	TCC203	*Myotis ruber*	Brazil
KT305793	TCC417	*Thyroptera tricolor*	Brazil
KT305795	TCC642	*Carollia perspicillata*	Brazil
GQ140352	TryCC507	*Carollia perspicillata*	Brazil
KT305794	TCC640	*Carollia perspicillata*	Brazil
KT305796	TCC1338	*Carollia perspicillata*	Venezuela
KP197175	CBT149	*Philander opossum*	Brazil
GQ140356	TryCC85	*Homo sapiens*	Brazil
KT305818	CanIII	*Homo sapiens*	Brazil
JN040972	TCC337	*Saguinus fuscicollis*	Brazil
AJ620269	VINCH_89	*Triatoma infestans*	Chile
GQ140355	TryCC844	*Homo sapiens*	Brazil
KT305817	M6241_cl6	*Homo sapiens*	Brazil
KT305815	TCC2557	*Phyllostomus hastatus*	Brazil
GQ140353	Y	*Homo sapiens*	Brazil
GQ140354	TryCC33	*Triatoma infestans*	Brazil
AJ620270	B7	*Phyllostomus discolor*	Brazil
GQ140363	TryCC495	*Carollia perspicillata*	Brazil

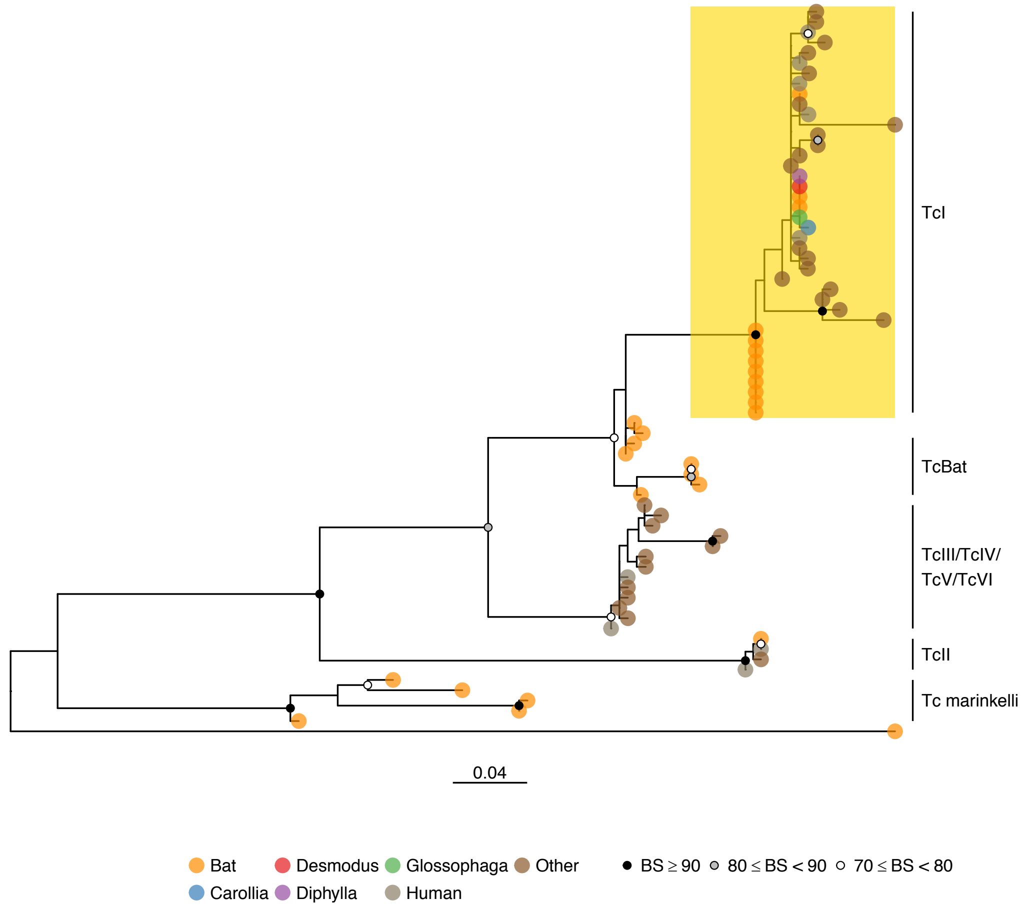

Figure S1. *Trypanosoma cruzi* cytB maximum likelihood phylogeny including novel sequences from Neotropical bat saliva. The phylogeny was constructed in RAxML based on a 476bp alignment of 71 *Trypanosoma* cytochrome B sequences. The phylogeny is rooted using *Trypanosoma dionisii* (Genbank accession FJ900249) as an outgroup and the TcI lineage is highlighted in gold. Novel sequences from Neotropical bat saliva are shown in blue (*C. perspicillata*), purple (*D. ecaudata*), green (*G. soricina*) and red (*D. rotundus*). Sequences from bats from other studies are colored in orange, sequences from humans are colored in gray, and sequences from any other host or vector species are colored in brown.

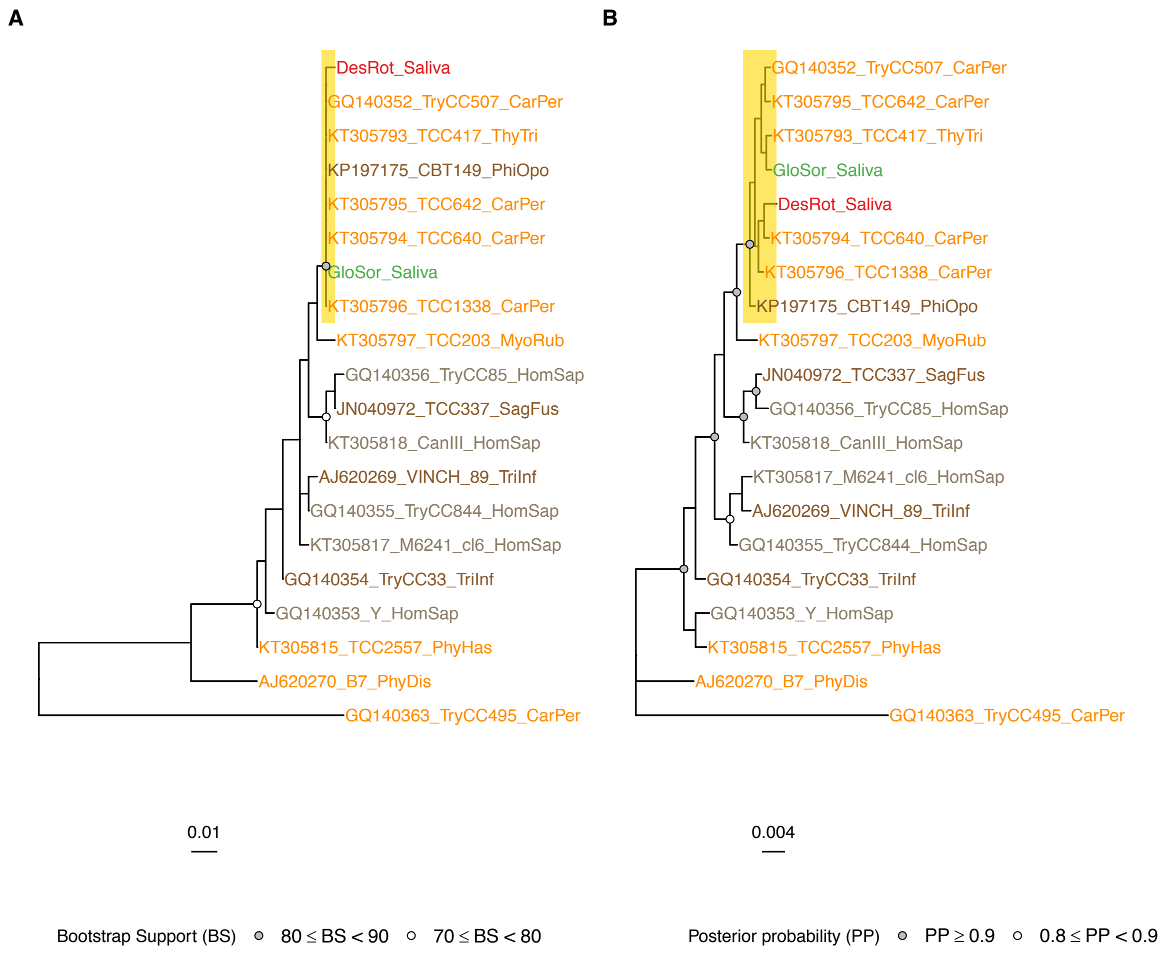

Figure S2. *Trypanosoma cruzi* gGAPDH phylogenies including novel sequences from Neotropical bat saliva. Phylogenies are based on a 517bp alignment of 20 *Trypanosoma* glycosomal glyceraldehyde 3-phosphate dehydrogenase sequences and were constructed using (A) Maximum Likelihood in RAxML and (B) Bayesian analysis in MrBayes. The phylogeny is rooted using *Trypanosoma dionisii* (Genbank accession GQ140363) as an outgroup, with the TcI lineage highlighted in gold. Novel sequences from Neotropical bat saliva are shown in green (*G. soricina*) and red (*D. rotundus*). Sequences from bats from other studies are colored in orange, sequences from humans are colored in gray, and sequences from any other host or vector species are colored in brown.
